# Cigarette Smoking and the Risk of Adult Myeloid Disease: A Meta-Analysis

**DOI:** 10.1371/journal.pone.0137300

**Published:** 2015-09-04

**Authors:** Peng Wang, Huifang Liu, Ting Jiang, Julun Yang

**Affiliations:** 1 Department of Pathology, Medical Faculty, Kunming University of Science and Technology, Kunming, P.R. China; 2 Department of Pathology, Kunming General Hospital, Clinical College of Kunming Medical University, Kunming, P.R. China; 3 Department of Pathology, Kunming General Hospital, Kunming, P.R. China; Queen's University Belfast, UNITED KINGDOM

## Abstract

**Background:**

The adult myeloid diseases, myelodysplastic syndrome and acute myeloid leukemia, have been reported to be associated with cigarette smoking, but the results have been conflicting. Previous studies may have ignored the relationship between myelodysplastic syndrome and acute myeloid leukemia, where approximately one-third of myelodysplastic syndrome cases will progress to acute myeloid leukemia, which could induce a serious bias in independent analyses. For the purposes of researching pathogenesis, we suggest that myelodysplastic syndrome and acute myeloid leukemia should be regarded as a single class of adult myeloid disease, and herein assessed the relationship between cigarette smoking and the risk of adult myeloid disease.

**Methods:**

The PubMed, Cochrane Library, EBSCO, and EMBASE databases were systematically searched for reports published from 1990 to 2015. Two authors independently assessed the methodological quality and the extracted data. The odds ratios and adjusted odds ratios (OR), a sensitivity analysis, and the publication bias were analyzed using the CMA v2 (Comprehensive Meta Analysis Version 2) software program.

**Results:**

Twenty-five studies were included in this meta-analysis. The publication dates ranged from 1990 to 2014. The pooled OR in current smokers and ever-smokers showed an increased risk of adult myeloid disease, with ORs of 1.45 (95% CI, 1.30–1.62; *p*<0.001) and 1.23 (95% CI 1.15–1.32; *p*<0.001) versus non-smokers, respectively. In the subset analyses, the OR of adult myeloid disease was increased regardless of the form of disease, geographical region, NOS (Newcastle Ottawa Scale) score, and source of controls. The smoking status was divided into <20 and ≥20 cigarettes per day, and these groups had ORs of developing adult myeloid disease of 1.24 (95% CI, 1.09–1.40; *p* = 0.001) and 1.32 (95% CI, 1.14–1.53; *p*<0.001), respectively. In the groups divided based on the number of years the subjects had smoked (<20 and ≥20 years), the ORs were 1.05 (95% CI, 0.90–1.23; *p* = 0.25) and 1.30 (95% CI, 1.16–1.45; *p*<0.001), respectively. Similarly, <20 and ≥20 pack-years were associated with ORs of 1.15 (95% CI, 1.03–1.29; *p* = 0.017) and 1.34 (95% CI, 1.18–1.52; *p*<0.001), respectively.

**Conclusions:**

This meta-analysis, for the first time, combined myelodysplastic syndrome with acute myeloid leukemia to assess the overall risk of adult myeloid disease, and it demonstrated that cigarette smoking is associated with a significantly increased risk of adult myeloid disease.

## Introduction

Adult myeloid diseases are a group of clonal diseases that affect the stem cells in bone marrow. Among the adult myeloid diseases, myelodysplastic syndrome (MDS) and acute myeloid leukemia (AML) have high incidence rates of 4 and 3.7 per 100,000 people, respectively. Moreover, the number of MDS cases reaches 40–50 per 100,000 in patients older than 70 years old [1, 2]. The median age at diagnosis is approximately 67 and 76 years for AML and MDS, and the conditions tend to occur earlier in Asian populations [2–5]. In addition, men have a significantly higher incidence rate than women [4, 5]. A striking feature of MDS is its genetic instability, and approximately one-third of MDS cases result in AML [2, 6–10]. The pathogenesis of MDS or AML is uncertain, particularly why MDS can transform into AML.

Tobacco is an established factor that can cause carcinogenicity, teratogenicity and mutagenicity. Up to half of current smokers will eventually die of a tobacco-related disease [11]. The monograph by the International Agency for Research on Cancer (IARC), the National Comprehensive Cancer Network Clinical Practice Guidelines (NCCN Guidelines) and the European Society for Medical Oncology Practice Guidelines (ESMO Guidelines) reported that adult myeloid disease is linked to smoking [3, 10, 12]. Attributable risk calculations suggest that about 15% of all leukemia deaths and 24% of myeloid leukemia deaths are accounted for as a result of cigarette smoking[13].

Since the strongest evidence comes from cohort studies, some data were also available from case-control studies. Early in 1978, Paffenbarger [14] found that cigarette smoking was related to AML. Many subsequent case-control studies focused on investigating the relationship between cigarette smoking and the risk of AML or MDS, but the results had significant bias [15–18]. Fircanis [19] evaluated the relationship between smoking and AML, and Tong [20] investigated the relationship between smoking and MDS. However, neither of these researchers examined the interrelationship between MDS and AML, wherein approximately one-third of MDS cases transform into AML. Independent analyses of AML or MDS alone cannot accurately assess the association with smoking. In fact, both AML and MDS show identical cellular morphology, clonal hyperplasia of myeloblast. The number of myeloblasts is considered as one of important differential features between them. Myeloblasts are less than 20% in MDS, nevertheless more than 20% in AML [21]. We thought that MDS and AML should be grouped together as a single class of “adult myeloid disease” for the purposes of researching pathogenesis, and we herein assessed the relationship between cigarette smoking and the risk of developing adult myeloid disease.

## Materials and Methods

### Literature search and study selection

A systematic literature search was conducted by two independent reviewers (Wang and Liu) of the PubMed, Cochrane Library, EMBASE and EBSCO databases for papers published from 1990 to 2015. The following search terms were used: (1) “AML” OR “acute myeloid leukemia” or “myelodysplastic syndrome” OR “MDS” OR “myelodysplastic” OR “myelodysplasia” OR “preleukemia”; (2) “smoking” OR “tobacco” OR “cigarette”. These search themes were combined using the Boolean operator “and” in several combinations without restrictions. In addition, the references reported in the identified studies were used to complete the search.

Studies eligible for inclusion in this meta-analysis met the following criteria: (1) the study design was a case-control study; (2) investigated the association between smoking and the risk of AML or MDS; (3) the case and control of studies must have been based on adults; (4) the diagnoses of AML and MDS were confirmed either histologically, pathologically, cytologically or by detailed medical records; (5) the odds ratios (OR) and the number of events could be calculated from the data presented;(6) published as a full text document in English. Two reviewers (Liu and Jiang) determined the study eligibility independently. Disagreements were resolved by consensus.

### Data extraction and quality assessment

Two investigators (Liu and Jiang) read the full text of the manuscripts independently and extracted the following data from each eligible study: first author’s name, publication year, country of origin, study period, source of controls, sample size, method of ascertainment of smoking, method of ascertainment of adult myeloid disease and the Newcastle Ottawa Scale (NOS) score [22]. The NOS score usually is used for assessing the quality of nonrandomized studies (case-control and cohort studies) in meta-analyses. To assess the quality of these studies, two reviewers (Liu and Jiang) independently read and scored each study according to the NOS [22]. NOS scores of 1–3, 4–6, and 7–9 were considered to indicate low, medium and high quality, respectively.

### Statistical analysis

We calculated the pooled odds ratios (OR) with the 95% CI by using the Comprehensive Meta-Analysis software program (Version 2.2.064 July 27, 2011). The statistical heterogeneity within studies was evaluated using a χ^2^-based Cochran’s Q statistic [23] and was further quantified using *I* ^*2*^ statistics (*I* ^*2*^ = 0–25%, no heterogeneity; *I* ^*2*^ = 25–50%, moderate heterogeneity; *I* ^*2*^ = 50–75%, large heterogeneity; and *I* ^*2*^ = 75–100%, extreme heterogeneity. For values of *I* ^*2*^ ≤ 50%, the fixed-effects model was used; *I* ^*2*^ ≥ 50%, the random-effects model was used) [23]. To investigate the potential heterogeneity, we also conducted a subgroup analysis based on the following four aspects: forms of disease, geographical region, source of controls and the NOS score. Publication bias was examined by Egger’s regression and Begger’s funnel plot (p ≤ 0.05 was considered to indicate the presence of significant publication bias) [24] and was adjusted by Duval and Tweedie’s trim-and-fill method [25]. A sensitivity analysis was performed to evaluate the potential effects of the removal of one study in each turn. If the outcome was significantly changed after one study was removed, then the study was excluded from the included studies due to selection bias, and a new analysis was conducted.

## Results

### Literature search

**Fig 1:**
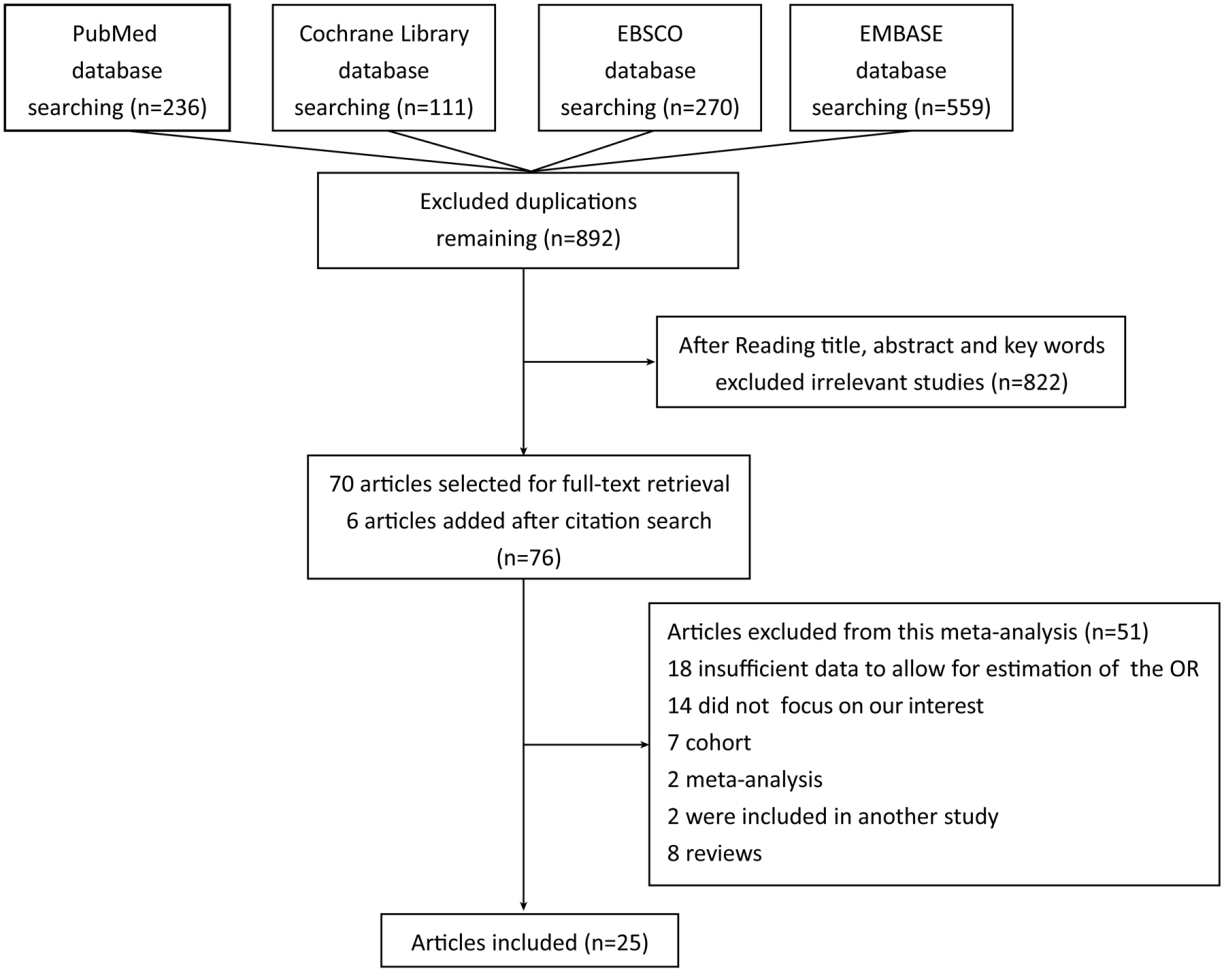
A flowchart of the study selection process.

There were twenty-five articles included in our meta-analysis [15–18, 26–46]. Two reviewers assessed seventy-six articles independently and excluded fifty-one articles. The reasons for exclusion of the articles were as follows: insufficient data to allow for estimation of the OR for eighteen studies, fourteen studies did not focus on our topic of interest, seven were based on cohort studies, ten were meta-analyses or reviews, and two were included in another article. Our search flow is shown in Fig 1.

### Characteristics of the included studies

The main characteristics of the included studies are shown in Table 1. Studies were published from 1990 to 2014. There were ten [16–18, 30, 32–35, 40, 41] studies performed in Europe, ten [26–29, 36–39, 45, 46] in North America and five [15, 31, 42–44] performed in Asia. A total of 8,074 adult myeloid disease cases and 31,805 controls were included in our meta-analysis. The controls of twenty-five studies came from different sources: fourteen [15, 16, 18, 26–29, 33, 34, 36, 38, 41, 45, 46] were population-based, ten [30–32, 35, 37, 39, 40, 42–44] were hospital-based, and only one [17] came from both a population and hospital. Cases in all included studies were confirmed mainly according to one method or several methods combined based on medical records, pathological and cytogenetic findings. Smoking factors were evaluated using the medical records in six [18, 27, 31, 35, 37, 44] studies, questionnaires in three [15, 39, 46] studies, personal interviews in twelve [16, 17, 28, 30, 32, 34, 36, 40–43, 45] studies, telephone interviews in three [26, 29, 33] studies and one [38] study included both a questionnaire and telephone interview. The data about matching were extracted from all of the included studies. According to the Newcastle-Ottawa Scale quality assessment system, sixteen [16, 17, 27–29, 34–36, 38–43, 45, 46] studies were high quality (NOS score ≥7), eight [15, 18, 26, 30, 32, 33, 37, 44] studies were medium quality (NOS score ≥4 and ≤6) and one study [31] was low quality (NOS = 3). Overall, the average score of all included studies was 6.68.

**Table 1:** Characteristics of the included studies.

Study	Year	Country	Period	Groups	Source of controls	Age (years)	No. of subjects	Adult myeloid disease confirmation	Smoking evaluation	Matching	NOS
Wilson	2014	UK	1990–2009	Control	PB	20–79	3417	Medical records	Medical records	Age, sex, index date	6
				MDS			849				
Musselman	2013	USA	2005–2009	Control	PB	20–79	692	Cytogenetic results integrated with pathologically confirmed	Self-administered questionnaire	Age, sex, and BMI	9
				AML			414				
Strom	2012	USA	2003–2007	Control	PB	18–80	636	Pathologically confirmed	Personal interviews	Age, sex, race, county of residence	7
				AML			638				
Kim	2012	Korea	1997–2008	Control	HB	50.5±16.9	1700	Pathologically confirmed	Medical records	Age, sex	5
				AML			415				
Lv	2011	China	2003–2006	Control	HB	≥18	806	Pathologically confirmed	Face-to-face interviews	Age, sex	7
				MDS			403				
Wong	2009	China	2003–2007	Control	HB	≥18	1444	Pathologically and cytogenetic confirmed	Personal blinded interviews	Age, sex	8
				AML			722				
Bjork	2009	Sweden	2001–2004	Control	PB&HB	≥20	278	Medical records	Face-to-face interviews	Age, sex, county of residence	8
				AML			104				
				MDS			75				
Richardson	2008	Germany	1986–1998	Control	PB	53.5±14	266	Medical records	Face-to-face interviews	Year of birth, sex, region	8
				ANLL			120				
Pekmezovic	2006	Serbia, Montenegro	2000–2003	Control	HB	20–85	160	Pathologically confirmed supplemented by medical records	Personal interviews, supplemented by medical records	Age, sex	7
				MDS			80				
Kasim	2005	Canada	1994–1997	Control	PB	20–74	5039	Pathologically confirmed	Mailed questionnaires with telephone follow-up	Age, sex, body mass index	8
				AML			307				
Strom	2005	USA	1999–2003	Control	HB	24–89	452	Pathologically confirmed	Mailed questionnaires	Age, sex, ethnicity	7
				MDS			354				
Speer	2002	USA	1984–1993	Control	HB	Median age (65)	7107	Medical records	Medical records	Age, sex	5
				AML			604				
Pogoda	2002	USA	1987–1994	Control	PB	25–75	412	Pathologically confirmed	Non-blinded interviews	Birth year, sex, race	7
				AML			412				
Dalamaga	2002	Greek	1995–2000	Control	HB	44–85	84	Pathologically confirmed supplemented by cytogenetic	Medical records	Age, sex	7
				MDS			84				
Stagnaro	2001	Italy	1990–1993	Control	PB	20–74	1779	Pathologically confirmed	Blind interviews	Age, sex, area of residence, education level, type of interview	8
				AML			223				
Bjork	2001	Sweden	1976–1993	Control	PB	35–76	351	Pathologically confirmed supplemented by cytogenetic	Structured telephone interview	Age, sex, county of residence	6
				AML			333				
Nisse	2001	France	1991–1996	Control	PB	62–74	204	Pathologically confirmed	Personal interviews	Age, sex	7
				MDS			204				
Nagata	1999	Japan	1995–1996	Control	PB	20–74	830	Pathologically confirmed	Mailed questionnaire	Sex, region	6
				MDS			111				
West	1995	UK	NA	Control	HB	≥15	399	Pathologically confirmed	Personal interviews	Age, sex, region, hospital and year of diagnosis	4
				MDS			399				
Wakabayashi	1994	Japan	1981–1990	Control	HB	51.3±15.8	150	Medical records	Medical records	Age, sex	3
				ANLL			75				
Mele	1994	Italy	1986–1989	Control	HB	≥30	467	Medical records	Personal interviews	Age, education, residence	6
				AML			118				
Sandler	1993	USA/Canada	1986–1989	Control	PB	18–79	618	Pathologically confirmed	Telephone interviews	Age, sex, race, region	7
				AML			423				
Brown	1992	USA	1981–1984	Control	PB	≥30	745	Medical records	Personal interviews	Age, region, vital status	9
				ANLL			134				
Brownson	1991	USA	1984–1990	Control	PB	≥20	3641	Pathologically confirmed	Medical records	Age	7
				AML			367				
Severson	1990	USA	1984–1986	Control	PB	20–79	128	Pathologically confirmed	Telephone interviews	Age, sex, family income	5
				ANLL			106				

**Abbreviations:** No. of subjects, Number of subjects; MDS, Myelodysplastic Syndrome; AML, Acute Myeloid Leukemia; ANNL, Acute Nonlymphocytic Leukemia; NOS, Newcastle-Ottawa Scale; PB, Population-Based; HB, Hospital-Based; BMI, Body Mass Index.

### Meta-analysis

#### Current smokers

**Fig 2:**
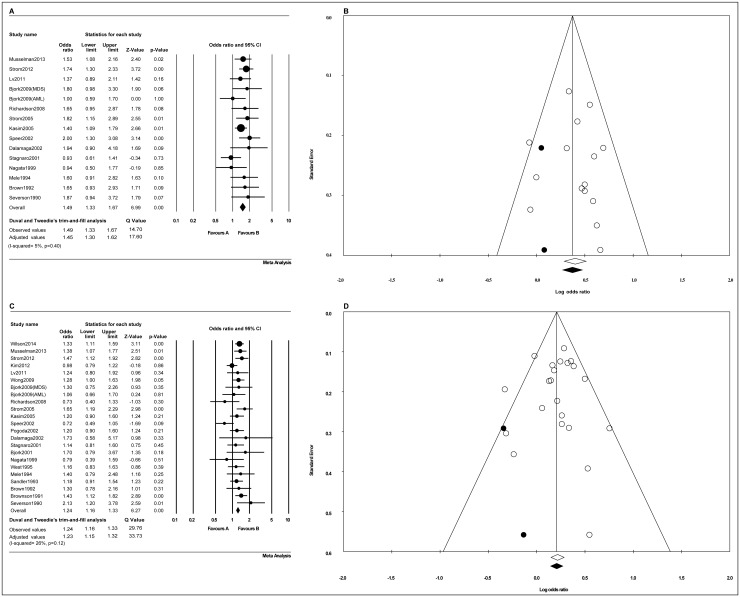
The odds ratio estimates of the risk of developing adult myeloid disease in smokers. (A) A forest plot of the risk of developing adult myeloid disease in current smokers. (B) Duval and Tweedie’s trim-and-fill funnel plots were used to observe and adjust the publication bias in current smokers. (C) A forest plot of the risk of developing adult myeloid disease in ever-smokers. (D) Duval and Tweedie’s trim-and-fill funnel plots were used to observe and adjust the publication bias in ever-smokers. Among Fig B and D, The white circles represent observed studies, and black circles represent possibly missed studies imputed using Duval and Tweedie’s trim-and-fill method. White and black rhombuses represent observed and theoretical combined effect size, respectively.

Thirteen [15, 16, 26, 28, 30, 35, 37–39, 41, 43, 45, 46] out of fourteen studies independently researched the relationship between MDS or AML and cigarette smoking, and one [17] study meanwhile researched MDS and AML. The pooled OR was 1.45 (95% CI, 1.30–1.62; *p*<0.001), and it was determined that current smokers had a 45% higher risk of developing adult myeloid disease compared with non-smokers, with low heterogeneity (Fig 2A). A funnel plot identified the presence of publication bias, and Duval and Tweedie’s trim-and-fill analysis was used to adjust the ORs (Fig 2B), with *p* = 0.46 in the Begg rank correction test and p = 0.89 in Egger’s linear regression test.

#### Ever-smokers

Twenty-one [15, 16, 18, 26–30, 32, 33, 35–39, 41–46] out of twenty-two studies independently researched the relationship between MDS or AML and cigarette smoking, and one study [17] meanwhile researched MDS and AML. The pooled OR was 1.23 (95% CI, 1.15–1.32; *p*<0.001), which confirmed that ever-smokers had a 23% increased risk of developing adult myeloid disease compared with non-smokers, with moderate heterogeneity (Fig 2C). A funnel plot identified the presence of publication bias, so Duval and Tweedie’s trim-and-fill analysis was used to adjust the ORs (Fig 2D), with *p* = 0.75 in the Begg rank correction test and *p* = 0.83 in Egger’s linear regression test.

A sensitivity analysis was conducted, and the pooled OR was not significantly changed in the current smokers (S1 Fig) or ever-smokers (S2 Fig) when individual studies were removed in each turn.

### Subgroup analysis

We performed subgroup analyses stratified by the forms of disease, geographical region, NOS score (high and medium quality studies) and the source of controls in this study (Table 2).

**Table 2:** The results of subgroup analyses performed according to the potential sources of heterogeneity.

Outcome	Form of disease		Geographical region			NOS score		Source of control		
	AML	MDS	Asia	Europe	Nor A	High	Medium	PB	HB	PB&HB
**Current smokers**										
No. of datasets	10	5	2	6	7	11	4	8	5	2
OR (95% CI)	1.49 (1.31–1.69)	1.52 (1.19–1.94)	1.22 (0.85–1.74)	1.31 (1.05–1.63)	1.62 (1.41–1.86)	1.47 (1.30–1.66)	1.62 (1.23–2.14)	1.44 (1.26–1.65)	1.71 (1.37–2.13)	1.32 (0.74–2.34)
*P value*	<0.001	0.001	0.29	0.02	<0.001	<0.001	0.001	<0.001	<0.001	0.35
*I* ^*2*^	18%	0%	0%	27%	0%	4%	23%	20%	0%	51%
Pub. bias	YES	YES	CBC	YES	YES	YES	NO	YES	NO	CBC
AOR (95% CI)	1.43 (1.27–1.61)	1.48 (1.17–1.86)		1.09 (0.90–1.31)	1.54 (1.36–1.75)	1.43 (1.27–1.61)		1.43 (1.25–1.63)		
**Ever-smokers**										
No. of datasets	16	7	4	9	10	15	8	13	8	2
OR (95% CI)	1.21 (1.12–1.31)	1.32 (1.16–1.50)	1.10 (0.95–1.28)	1.24 (1.10–1.40)	1.31 (1.19–1.44)	1.29 (1.19–1.41)	1.16 (0.94–1.43)	1.30 (1.19–1.41)	1.17 (0.97–1.41)	1.16 (0.81–1.66)
*P value*	<0.001	<0.001	0.21	<0.001	<0.001	<0.001	0.01	<0.001	0.10	0.429
*I* ^*2*^	37%	0%	19%	0%	47%	0%	60%	2%	51%	0%
Pub. bias	YES	NO	NO	NO	YES	NO	YES	NO	YES	CBC
AOR (95% CI)	1.20 (1.11–1.30)				1.29 (1.17–1.42)		1.13 (1.01–1.26)		1.06 (0.88–1.28)	

**Abbreviations:** No. of datasets, Number of datasets (papers); MDS, Myelodysplastic Syndrome; AML, Acute Myeloid Leukemia; Nor A, North America; NOS, Newcastle-Ottawa Scale; Pub. bias, Publication bias; CBC, cannot be calculated; OR, odds ratio; AOR, Adjusted OR; CI, Confidence interval; PB, Population-based; HB, Hospital-based.

#### Current smokers

**Fig 3:**
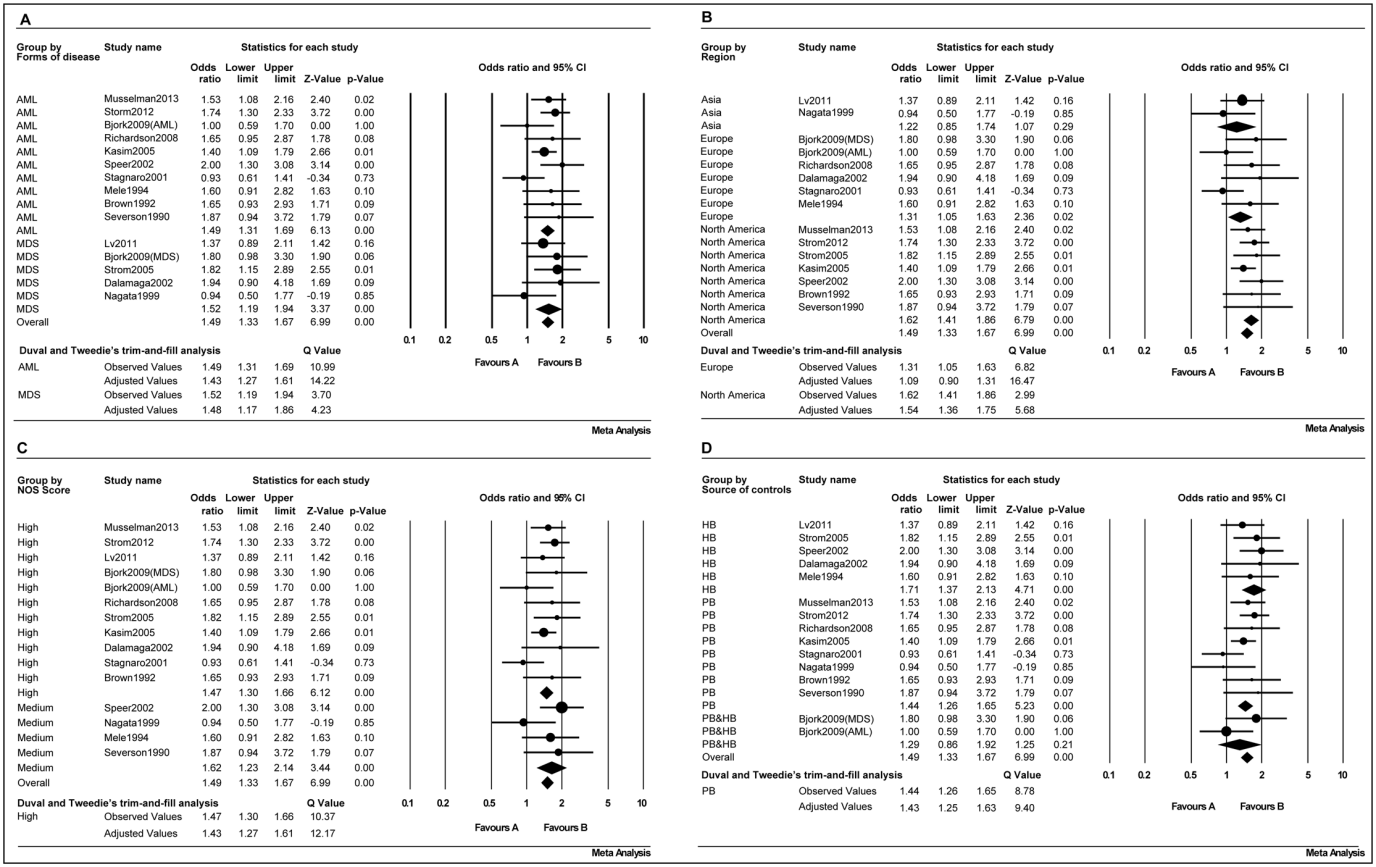
Forest plots of the risk of adult myeloid disease in current smokers. (A) Forms of disease, (B) geographical region, (C) NOS score (high and medium quality), (D) source of controls.

Group stratified by the form of disease showed a pooled OR of 1.43 (95% CI, 1.27–1.61; *p*<0.001) for AML and 1.48 (95% CI, 1.17–1.86; *p* = 0.001) for MDS (Fig 3A). When the subjects were grouped by region, the data showed that the pooled OR was 1.22 (95% CI, 0.85–1.74; *p* = 0.29) for Asia, 1.09 (95% CI, 0.90–1.31; *p* = 0.02) for Europe and was 1.54 (95% CI, 1.36–1.75; *p*<0.001) for North America (Fig 3B). In the data grouped by the NOS score, it was shown that the pooled OR was 1.43 (95% CI, 1.27–1.61; *p*<0.001) for high quality studies and was 1.62 (95% CI, 1.23–2.14; *p* = 0.001) for medium quality studies (Fig 3C). When the data were grouped based on the source of controls, the pooled OR was 1.43 (95% CI, 1.25–1.63; *p*<0.001) for population-based controls and was 1.71 (95% CI, 1.37–2.13; *p*<0.001) for hospital-based controls (Fig 3D).

#### Ever-smokers

**Fig 4:**
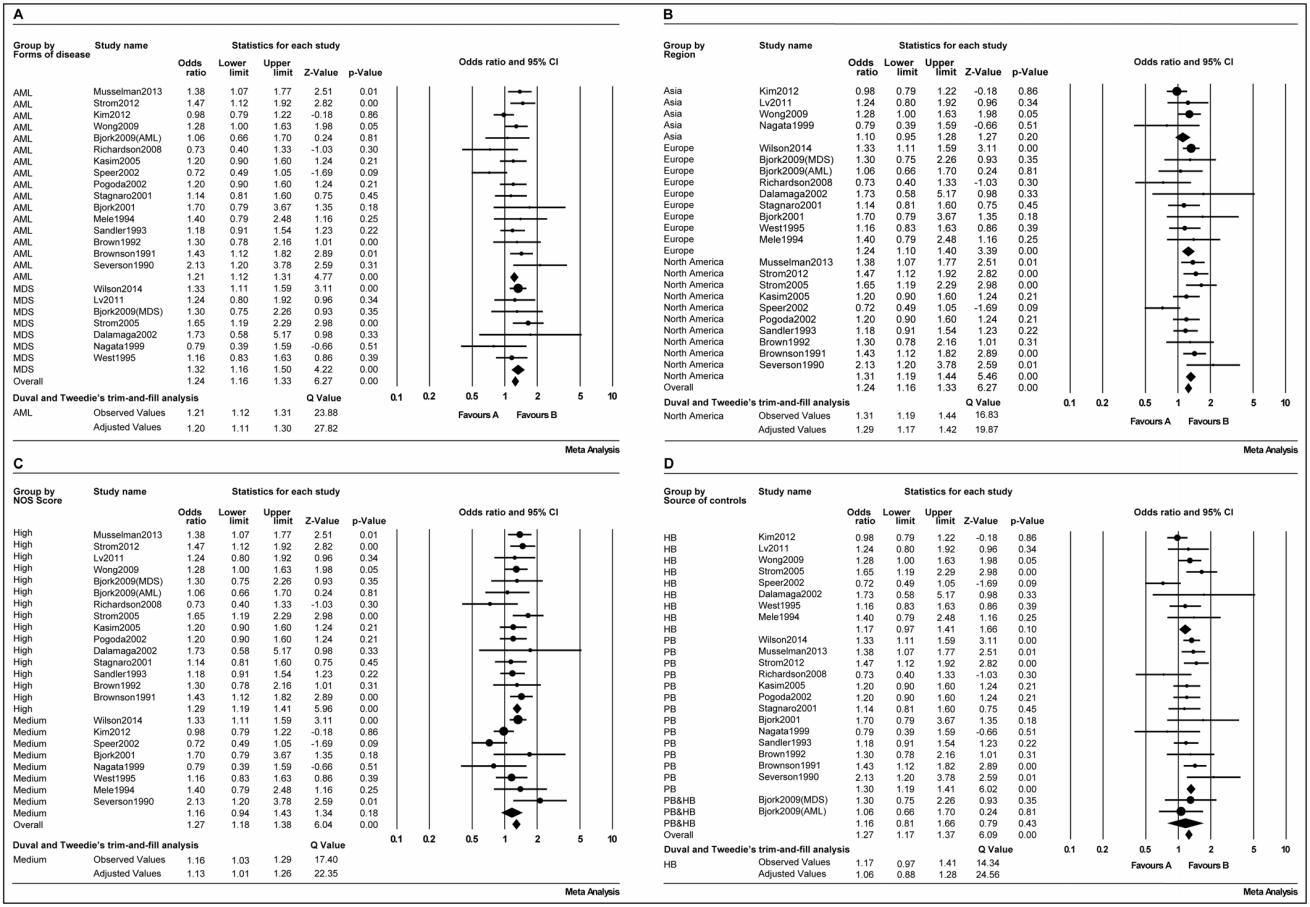
Forest plots of the risk of adult myeloid disease in ever-smokers. (A) Forms of disease, (B) geographical region, (C) NOS score (high and medium quality), (D) source of controls.

The subjects were also grouped based on the form of disease, geographical region, NOS score and source of control, all results indicated an significant increased OR, but a little less than current smokers. The results were shown in Table 2 and Fig 4.

### Smoking status analysis

**Fig 5:**
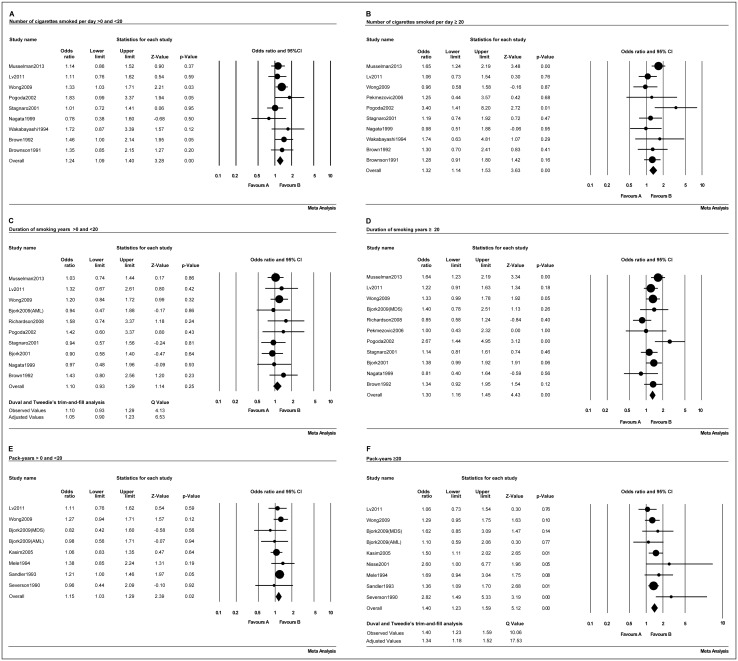
Forest plots of the association of adult myeloid disease with the smoking status based on cigarettes smoked per day (A-B), the duration of smoking (in years) (C-D), and the pack-years smoked (E-F).

Cigarette smoking does not exert its carcinogenic effects via a single event, but is instead a cumulative process. To further investigate the relationship between cigarette smoking and adult myeloid disease, three aspects were analyzed: the number of cigarettes smoked per day, the number of years of smoking and the pack-years smoked. The outcomes are shown in Table 3. Patients who smoked < 20 (Fig 5A) and ≥20 (Fig 5B) cigarettes per day had 1.24 (95% CI, 1.09–1.40; *p* = 0.001) and 1.32 (95% CI, 1.14–1.53; *p*<0.001) increased risks of developing adult myeloid disease, respectively. For patients with <20 (Fig 5C) and ≥20 (Fig 5D) years of smoking, the risk of adult myeloid disease was 1.05 (95% CI, 0.90–1.23; *p* = 0.25) and 1.30 (95% CI, 1.16–1.45; *p*<0.001), respectively. Similarly those with <20 (Fig 5E) and ≥20 (Fig 5F) pack-years had a risk of adult myeloid disease of 1.15 (95% CI, 1.03–1.29; *p* = 0.017) and 1.34 (95% CI, 1.18–1.52; *p*<0.001), respectively.

**Table 3:** Odds ratio estimates of the risk of adult myeloid disease according to the smoking status.

Outcome	Cigarettes per day		Duration of smoking (years)		Pack-years	
	>0 and<20	≥20	>0 and<20	≥20	>0 and<20	≥20
No. of datasets	9	10	10	11	8	9
OR (95% CI)	1.24(1.09–1.40)	1.32(1.14–1.53)	1.10(0.93–1.29)	1.30(1.16–1.45)	1.15(1.03–1.29)	1.40(1.23–1.59)
*p Value*	0.001	<0.001	0.25	<0.001	0.017	<0.001
*I* ^*2*^	0%	18%	0%	36%	0%	20%
Pub. bias	NO	NO	YES	NO	NO	YES
AOR			1.05(0.90–1.23)			1.34(1.18–1.52)

**Abbreviations:** No. of datasets, number of datasets; OR, Odds ratio; AOR, Adjusted OR; CI, Confidence interval; Pub. bias, Publication bias.

## Discussion

Recently, Fircanis [19] and Tong [20] analyzed association of smoking with AML and MDS by meta-analysis, respectively. Since approximately one-third of MDS cases could progress to AML, we supposed that the inclusion of some transformational cases (where the phenotype was AML but where the disease had developed from MDS) could have induced serious bias in independent analyses. Therefore, we conducted this meta-analysis that combined MDS with AML together as a single class of “adult myeloid disease” to eliminate bias.

To our knowledge, this study is the first meta-analysis that combined MDS with AML to assess the overall risk of adult myeloid disease due to cigarette smoking. We extracted twenty-five studies that included 8,074 cases and 31,805 controls, and demonstrated a statistically significant association between current smoking and an increased incidence of adult myeloid disease. Current smokers were shown to have a 45% higher risk of developing adult myeloid disease than non-smokers. Ever-smokers had a 23% higher risk of developing adult myeloid disease than non-smokers, indicating that current smokers had a higher risk of adult myeloid disease than ever-smokers. One possible reason was that current smokers might have a higher total cumulative dose such as nicotine, benzene and polycyclic aromatic hydrocarbon, and longer exposure time of smoking than ever smokers.

In addition, we found that the risk of myeloid disease had no significant drops after cessationin ever-smokers. As we known, tobacco is one of the most potent carcinogenic mixtures and the carcinogenic effect of cigarette smoking can persist for decades after smoking cessation. Few studies have reported on the dynamics and mechanism following cessation. Most recent two [47, 48] studies showed that DNA methylation levels in ever-smokers approached those of never smokers several years after smoking cessation. Therefore, the DNA methylation maybe one factor that the risk of myeloid disease had no significant drops after cessationin ever-smokers. The truly irreversible nature of smoking-related and associated biomarkers are needed to be investigated.

According to the sub-analysis of the smoking status, there was a dose-relationship between cigarette smoking and adult myeloid disease. First, the risk of adult myeloid disease increased from 24% to 32% when smokers smoked <20 vs.≥ 20 cigarettes per day. Second, the risk of adult myeloid disease increased from 5% to 30% in smokers who smoked <20 years compared with those who smoked ≥20 years. Finally, the risk of adult myeloid disease increased from 15% to 34% when the comparison was made of smokers who had smoked <20 vs.≥ 20 pack-years. Based on these results, we confirmed that there were close, positive, and cumulative effects of smoking on the development of adult myeloid disease. Moreover, we also found that cigarette smoking in North America was associated with a higher ORs than in Europe and Asia, but limited studies were inadequate for a clear conclusion that people who live in North America were susceptible population.

Now that cigarette smoking had a high risk to induce adult myeloid disease, Does it play a key role in MDS transformation? There have as yet been few detailed studies on the MDS transformation into AML by the individuals’ smoking habits, however, indirect studies may provide us related clues. Over the years, a large number of studies have been presented that indicate a causal link between MDS development and genetic abnormality, related genes liked *bcl-2* [49], *C-CBL* [50], *CD95* [51], *HOXA9* [52], *TNFα* [53]. In addition, CpG methylation [54, 55], telomere shortening [56, 57] and microsatellite instability [58–60] also were found association with MDS progression to AML. Most importantly all above mentioned factors were affected by cigarette smoking [61–70]. Based on these studies, it indirectly indicated that cigarette smoking may be an induced factor on MDS development.

Benzene of smoking is a known clastogen, causing chromosomal aberrations *in vitro*, and prolonged exposure to mean concentrations of >64 mg/m^3^ may be associated with chromosomal aberrations. In addition, increased risks of developing all types of leukemias were found at all concentrations of exposure, while exposure to levels above 40 ppm increased the risk of acute nonlymphocytic leukemia [71]. Zhang [72] conducted a chromosome-wide aneuploidy study (CWAS) in workers exposed to benzene, and their findings suggested that the development of aneuploidy may be a potential mechanism underlying benzene-induced leukemia. Benzene is mainly found in the atmosphere, and almost all human exposure is through inhalation (95% of daily intake) [71]. Personal exposure assessment research has indicated that an average cigarette smoker inhales 6–10 times the benzene inhaled by average non-smokers, and approximately 90% of a smoker’s benzene exposure is from smoking [73]. Consequently, the tobacco use may lead to chromosomal and karyotype aberrations due to increased benzene exposure, thus resulting in MDS or AML.

Our study is associated with some limitations that may have affected the results. First, the results were calculated from published studies, and our included studies cannot cover published studies that could have led to a publication bias. In addition, published studies are often positive, and the omission of unpublished studies (with negative findings) may lead to an overestimation of the pooled OR. Second, the methods used to evaluate the smoking habits were mainly questionnaires or reports, rather than based on determinations of the blood levels of cotinine. Third, some of the subset analyses, although specified *a priori*, were performed on small datasets. Finally, methodological differences and confounding factors in the included studies were unavoidable.

## Conclusion

In summary, we herein demonstrated that cigarette smoking leads to a significantly increased risk of adult myeloid disease in adults. Cigarette smoking does not act as a carcinogenic factor via a single exposure, and instead leads to a cumulative process. We found that there was a positive risk between smoking and adult myeloid disease when a threshold smoking dose was crossed. More and larger prospective studies should be performed, and future studies should focus on investigating the pathway(s) by which cigarette smoking can induce adult myeloid disease.

## Supporting Information

S1 Fig The results of sensitivity analyses in current smokers. (TIF)

S2 Fig The results of sensitivity analyses in ever-smokers. (TIF)

S1 PRISMA Checklist The PRISMA checklist. (DOC)
