# Supplementary figures and images for: Cigarette Smoking and the Risk of Adult Myeloid Disease: A Meta-Analysis
Source: PLoS One. 2015 Sep 4;10(9):e0137300. doi: 10.1371/journal.pone.0137300 (PMC4560392; doi:10.1371/journal.pone.0137300)

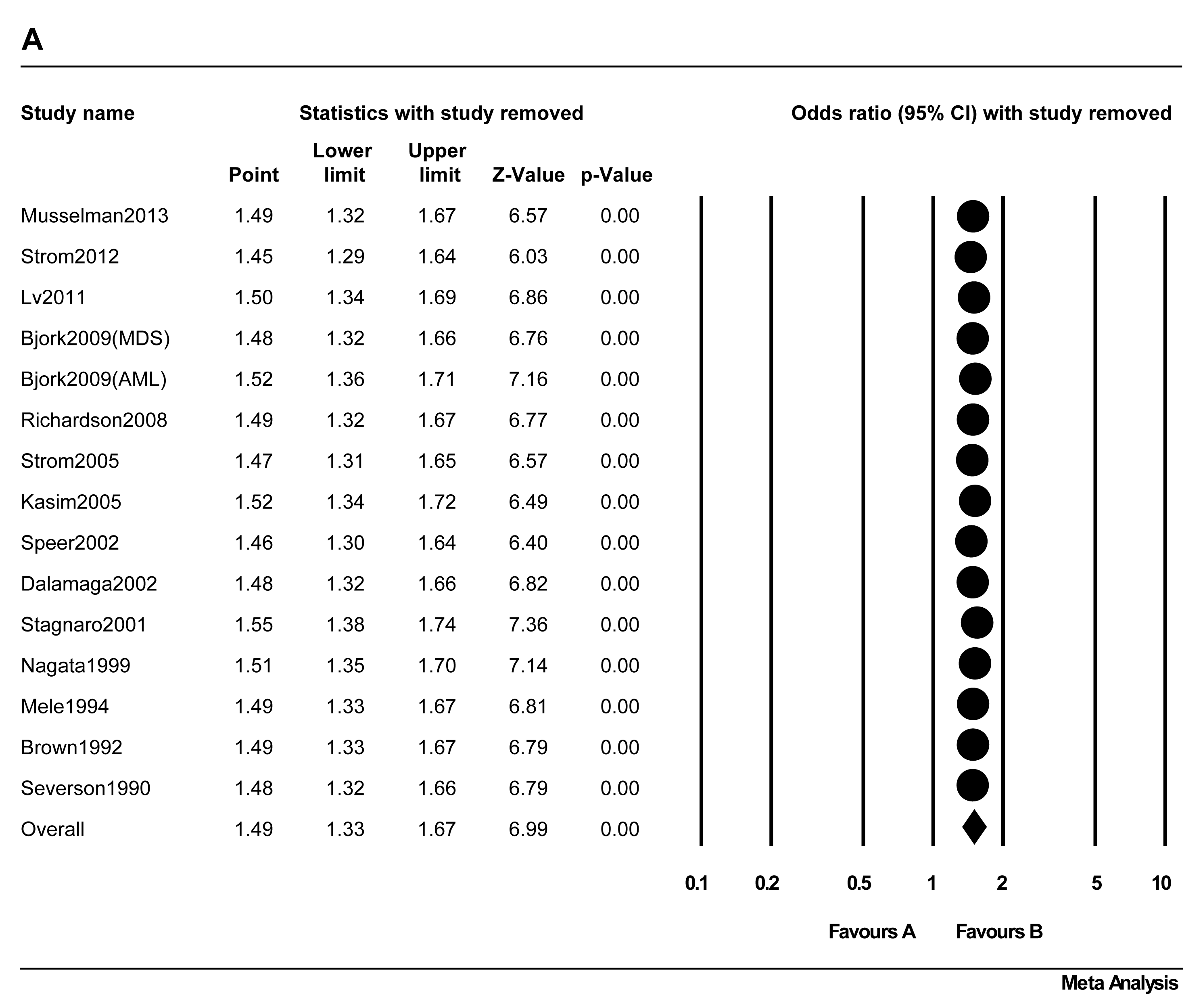

Supplement: S1 Fig — (TIF) [file pone.0137300.s001.tif]

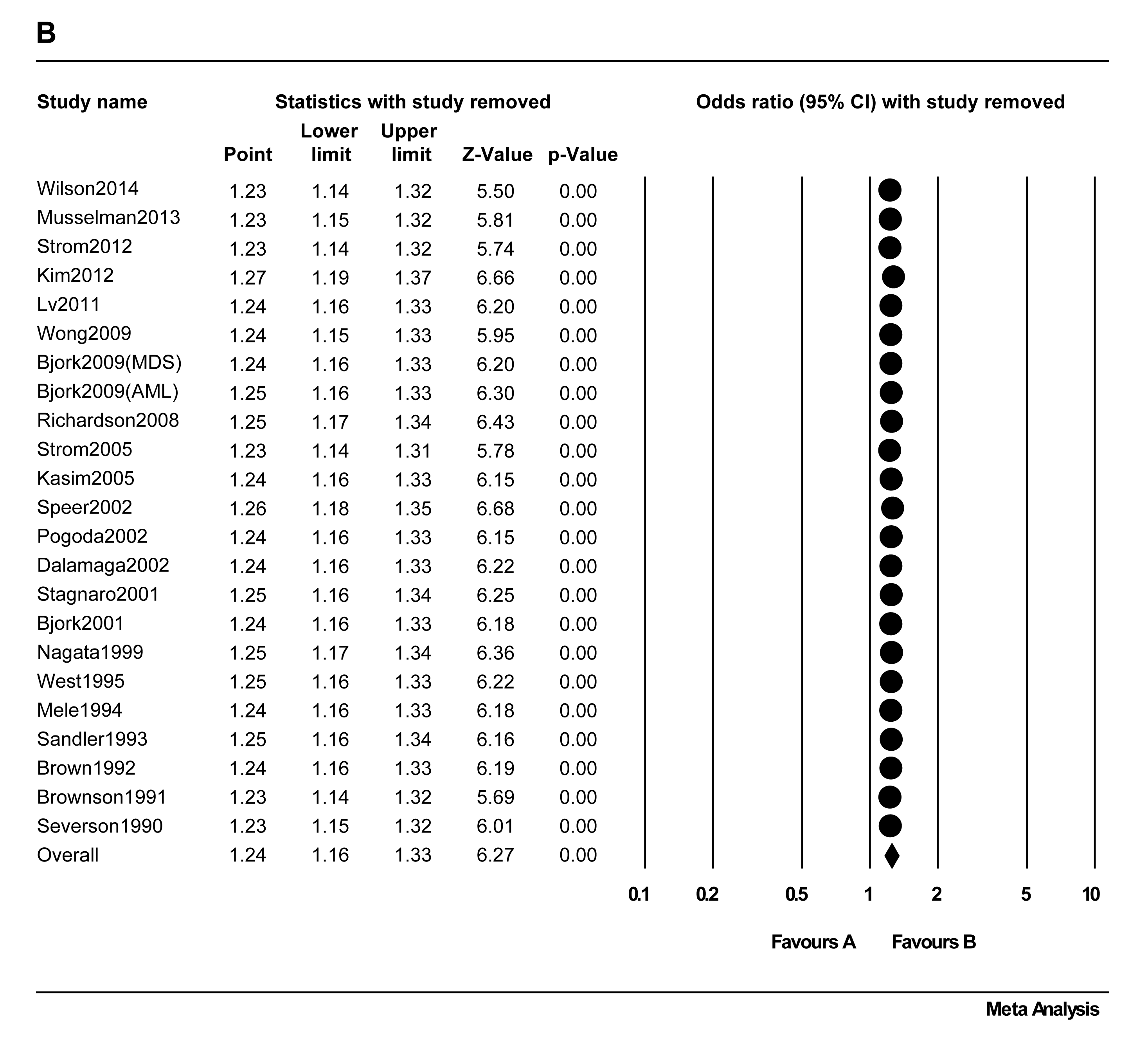

Supplement: S2 Fig — (TIF) [file pone.0137300.s002.tif]
